# The good, the bad, and the ugly: hyperprogression in cancer patients following immune checkpoint therapy

**DOI:** 10.1186/s13073-019-0661-7

**Published:** 2019-07-24

**Authors:** Erich Sabio, Timothy A. Chan

**Affiliations:** 1 Human Oncology and Pathogenesis Program, Memorial Sloan Kettering Cancer Center, New York, NY USA; 2 Immunogenomics and Precision Oncology Platform, Memorial Sloan Kettering Cancer Center, New York, NY USA; 3 Department of Radiation Oncology, Memorial Sloan Kettering Cancer Center, New York, NY USA

**Keywords:** Immune checkpoint, Hyperprogression, Cancer, T cells, Antigen, Mutation

## Abstract

Immune checkpoint blockade therapy can elicit robust and durable responses in a variety of cancer types. While many patients do not respond, recent reports highlight a distinct group of patients whose tumors undergo rapid growth, leading to progressive disease and poor outcome. In this perspective, we synthesize and summarize some important issues surrounding hyperprogression, defining characteristics, prognostic implications, and controversies.

## Immune checkpoint blockade-associated hyperprogression

While advances in immune checkpoint blockade (ICB) therapy have made transformative changes in oncologic care, both the lack of response in most patients and serious immune-related adverse events in a subset of patients have motivated the field to identify characteristics identifying patients who benefit from treatment. One serious outcome of ICB therapy has been described as tumor hyperprogressive disease (HPD). This phenomenon refers to the acceleration of tumor growth following ICB treatment that results not just from immune infiltration but from true disease progression. There are anecdotal and published descriptions of patient disease burden increasing by greater than 20–30-fold after only a few doses (or even one dose) of ICB. Although the existence of this phenomenon is controversial, physicians have observed it in practice, reporting that hyperprogression is unmistakable in extreme cases.

Early on, physicians treating patients with ICB noted that some patients appear to progress more rapidly. The reported incidence of this phenomenon ranges from a few percent to approximately 30%. Evidence of hyperprogression is found in the crossover of survival curves that is frequently seen in clinical trials comparing ICB to non-ICB treatments. Crossover of treatment arms is thought to demonstrate the presence of a subpopulation of patients who do worse on ICB compared to the respective control arm. While crossover does occur in non-ICB trials, some have argued that it is more frequent and pronounced in ICB-related trials. In considering HPD, it is critical that rigorous criteria, associated risk factors, and underlying biological mechanisms are defined as part of the effort to acknowledge and understand this phenomenon.

## Characteristics of hyperprogression and key considerations toward a consensus definition

While the exact set of clinical criteria for HPD varies between studies, the common requirement is a comparison of tumor growth shortly before and after immunotherapy is initiated. For example, an empirically derived threshold of a ≥ 2-fold increase in tumor volume change with respect to time, a metric that is referred to as tumor growth rate (TGR), segregated patients who had markedly accelerating disease and poor overall survival [1–3]. Though other studies define HPD using alternative thresholds in TGR [2], as well as alternative methods that include linear growth in tumor diameter [4, 5] or pace of progression and time-to-failure [6], no harmonized and universal definition of hyperprogression currently exists. Such rigorous definitions are critical for an unbiased evaluation of the frequency of hyperprogression. Future studies and rigorous multidisciplinary evaluation by tumor boards should assess the relative performance and optimal threshold for these criteria for identifying patients with HPD who will not benefit from continued treatment. Critically, pre-ICB treatment imaging and tumor kinetics are needed, and these data are limited. As the field refines these parameters, a key question will be to what extent we can approximate accelerated disease using empirically defined clinical metrics of progression while still being able to distinguish HPD from an intrinsically aggressive tumor. One important deficiency to resolve is the imbalance of pre-therapy imaging from patients with potential hyperprogression compared to that from control cohorts. Pre-ICB tumor growth kinetic data are crucial for establishing an accurate baseline. Furthermore, for any proposed criteria, it will be important to know whether the combination discriminates between hyperprogression and pseudoprogression (temporary enlargement of the tumor due to inflammatory infiltration) in a manner that is both accurate and timely.

## Risk factors

HPD has been shown to be associated with several putative risk factors, including age, genomic alterations, and metastasis burden, although the generalizability of these findings is currently unclear owing to limitations in cohort size and composition. For example, in a cohort of 131 patients across multiple tumor types, the 12 patients with HPD were significantly older, while age was correlated with tumor growth as defined by Response Evaluation Criteria in Solid Tumors (RECIST) criteria [1]. To accurately assess patient risk and benefit, future studies that examine these preliminary findings need to control for tumor type, stage, and other clinical covariates, particularly in studies that examine these same covariates and their association with response or resistance to immunotherapy. In a similar study of 102 patients with multiple cancer types, alterations in EGFR were predominant while MDM2/MDM4 amplifications were exclusive to six patients with HPD [6]. While other studies have also noted MDM2/MDM4 alterations in select HPD patients [7, 8], this is not the case in non-small cell lung carcinoma (NSCLC) [9]. Some investigators have found that regional recurrence in head and neck squamous cell carcinoma (HNSCC) [4] or more than two metastatic sites in NSCLC [2] prior to therapy are associated with a higher incidence of HPD. Regional recurrence in HNSCC and metastatic disease in most other cancer types are associated with a poor prognosis, because the tumors have successfully acquired the necessary traits for survival in the primary lesion and metastasis to distant sites. Therefore, it can be difficult to disentangle the relative effects of prognostic versus predictive factors. Ultimately, while the prospect of identifying predictive correlates that potentially either spare or curtail disease progression in patients at risk for developing HPD is attractive, it is important to address, and control for, the degree to which these correlates are simply prognostic for the patients represented in an HPD subgroup. Additional studies are necessary to validate and determine the breadth of tumor histologies for which each risk factor is predictive.

## Biological mechanisms underlying hyperprogression

A handful of biological mechanisms have been proposed to explain HPD. In a study of 152 NSCLC patients, where 25% of a cohort of 152 NSCLC patients had HPD, differences in the immune infiltrate were assessed between a small subset of the HPD vs non-HPD patients [9]. Hyperprogression was directly correlated with pre-existing myeloperoxidase (MPO)^+^ myeloid cells, and inversely correlated with programmed cell death-ligand 1 (PD-L1) expression on tumor cells. Furthermore, the presence of a CD163^+^CD33^+^PD-L1^+^ M2-like macrophage population with a distinct epithelioid morphology and clustering phenotype was always present, and nearly exclusive to the subset of HPD patients analyzed, suggesting that these macrophages are important in the etiology of HPD. Curiously, in T cell-deficient xenograft mouse models that recapitulate HPD, accelerated tumor growth was dependent on both the Fc region of anti-programmed cell death 1 (PD-1) and macrophages, suggesting that the Fc region of anti-PD-1 signals through macrophage Fc receptors to activate pro-tumorigenic or immunosuppressive pathways. These results suggest that it is important to consider the interaction between the distinct Fc regions used in different PD-1 antibodies, or the distinct Fc receptor variants of each patient, which have been shown to affect binding affinity or response in other antibody therapies. While these results are fascinating, HPD only occurred in three of the six xenograft and patient-derived xenograft models tested, all of which had similarly low levels of PD-1^+^ cells, suggesting that tumor cell characteristics may also influence the potential to develop HPD.

Another study reported that PD-1 was highly expressed in the tumor cells of a patient who developed HPD, as well as in the BALB/c-derived mouse lung carcinoma line M109 [10]. The authors reported that anti-PD-1 treatment promotes tumor growth of M109 cells injected into NSG mice or in culture, and that PD-1 expression on the tumor itself is a critical factor in vivo. Kamada et al. [7] observed that HPD occurred in 10% of gastric cancer patients treated with anti-PD-1 antibody. Gastric cancer patients with HPD had tumor infiltrating effector Treg (eTreg) cells that were FoxP3^high^CD45RA^−^CD4^+^. These tumor infiltrating cells were abundant and highly suppressive in tumors, and expressed PD-1. After treatment with anti-PD-1, these eTregs became highly activated in HPD patients, and PD-1 blockade significantly enhanced in vitro Treg suppressive activity. Genetic or therapeutic perturbation of PD-1 in Treg cells in mice recapitulated the enhanced proliferative capacity and immunosuppressive function in eTregs. Thus, PD-1 blockade may promote the proliferation of highly suppressive PD-1^+^ eTregs in HPD tumors, leading to inhibition of anti-tumor immunity [7].

## Conclusions

Do we think hyperprogression due to ICB treatment exists? In short, yes. The senior author of this Comment has personally witnessed tremendously explosive growth of tumor load within only a few weeks of receiving ICB therapy, consistent with a rapid kinetic profile that far outstripped the pretreatment growth rate. In some of these cases, cessation of previous therapies was not a confounding factor. There is no question that this phenomenon exists (at least at the time of this perspective, given available data). Do we know the true frequency of HPD? The answer here is no. Owing to the various uncertainties of measuring progression inherent in the current crop of studies and the relatively low numbers of patients examined so far, we cannot be certain of how frequently HPD occurs.

To gain greater knowledge about HPD, certain efforts can be made. Most prospective ICB trials have inadequate imaging data prior to treatment with immunotherapy. It would be useful to include closer scanning schedules during this period because this will allow better characterization of pre-ICB tumor kinetics. Additionally, trial correlatives should include the biomarkers discussed above that are potentially linked to HPD. The primary challenge is to develop harmonized criteria for defining HPD and to develop steps for clinical implementation. Other critical issues that need to be resolved include a lack of validated predictive factors, knowledge of how tumor microenvironmental and tumor cell intrinsic factors collaborate to influence HPD, and whether the different approved immune checkpoint agents have different potential for causing HPD. What is clear is that we need to study this issue in depth and we need to do so quickly to enable us to “first do no harm.”
